# Assessment of the abstract reporting of systematic reviews of dose-response meta-analysis: a literature survey

**DOI:** 10.1186/s12874-019-0798-5

**Published:** 2019-07-15

**Authors:** Peng-Li Jia, Bin Xu, Jing-Min Cheng, Xi-Hao Huang, Joey S. W. Kwong, Yu Liu, Chao Zhang, Ying Han, Chang Xu

**Affiliations:** 1 School of Management, Shanxi Medical University, Taiyuan, 030619 China; 2 West China School of Public Health, NO.4 West China Hospital, Sichuan University, Chengdu, China; 3 West China School of Medicine, West China Hospital, Sichuan University, Chengdu, China; 4 JC School of Public Health and Primary Care, Faculty of Medicine, The Chinese University of Hong Kong, Sha Tin, Hong Kong; 5 Gansu Provincial Maternity and Child-care Hospital, Gansu, China; 6 Center for Evidence-Based Medicine and Clinical Research, Taihe Hospital, Hubei University of Medicine, Shiyan, China; 7 Chinese Evidence-based Medicine Center, West China Hospital, Sichuan University, Chengdu, China

**Keywords:** Systematic review, Dose-response meta-analysis, Abstract reporting, Literature survey

## Abstract

**Background:**

There is an increasing number of published systematic reviews (SR) of dose-response meta-analyses (DRMAs) over the past decades. However, the quality of abstract reporting of these SR-DRMAs remains to be understood. We conducted a literature survey to investigate the abstract reporting of SR-DRMAs.

**Methods:**

Medline, Embase, and Wiley online Library were searched for eligible SR-DRMAs. The reporting quality of SR-DRMAs was assessed by the modified PRISMA-for-Abstract checklist (14 items). We summarized the adherence rate of each item and categorized them as well complied (adhered by 80% or above), moderately complied (50 to 79%), and poorly complied (less than 50%). We used total score to reflect the abstract quality and regression analysis was employed to explore the potential influence factors for it.

**Results:**

We included 529 SR-DRMAs. Eight of 14 items were moderately (3 items) or poorly complied (5 items) while only 6 were well complied by these SR-DRMAs. Most of the SR-DRMAs failed to describe the methods for risk of bias assessment (30.2, 95% CI: 26.4, 34.4%) and the results of bias assessment (48.8, 95% CI: 44.4, 53.1%). Few SR-DRMAs reported the funding (2.3, 95% CI: 1.2, 3.9%) and registration (0.6, 95% CI: 0.1, 1.6%) information in the abstract. Multivariable regression analysis suggested word number of abstracts [> 250 vs. ≤ 250 (estimated ß = 0.31; 95% CI: 0.02, 0.61; *P* = 0.039)] was positively associated with the abstract reporting quality.

**Conclusion:**

The abstract reporting of SR-DRMAs is suboptimal, substantial effort is needed to improve the reporting. More word number may benefit for the abstract reporting. Given that reporting of abstract largely depends on the reporting and conduct of the SR-DRMA, review authors should also focus on the completeness of SR-DRMA itself.

**Electronic supplementary material:**

The online version of this article (10.1186/s12874-019-0798-5) contains supplementary material, which is available to authorized users.

## Background

Systematic reviews (SRs) and meta-analyses are powerful evidence in guiding health policies and informed decision-making [1–3]. Appropriate reporting of SRs and meta-analyses is thus vital for the effective utilization of high-quality evidence in healthcare. This prompted the development of guidelines and checklists for standardized reporting, such as the well-known Preferred Reporting Items for Systematic Reviews and Meta-Analyses (PRISMA) statement [4].

Reporting of abstracts of published SRs and meta-analyses has been highlighted in previous literatures [5, 6]. An abstract summarizes the contents of a research report, in this case a SR or meta-analysis, in a brief pattern for users to outline the research evidence, and it is often the only freely accessible information for some users of evidence. Well-reported abstracts are essential in assessing the study validity, clarifying the applicability of results, and facilitating the peer-reviewing process [6, 7]. Literature surveys on abstracts reporting of SRs however demonstrated that the overall abstract reporting was suboptimal that the completeness of information was insufficient [8]. Great efforts were taken to improve the quality of abstract reporting for SRs, for example, the PRISMA for Abstracts statement released in 2013, as an extension of the PRISMA statement [7].

Dose-response meta-analysis (DRMA) is a meta-analysis that explores the dose-response relationship between continuous (or discrete) independent (e.g. sleep duration) and dependent variable (e.g. risk of death) [9]. Unlike traditional meta-analysis, DRMA allows investigators to determine whether there are different effects for presence and absence of exposure (or intervention), as well as whether the effects varying according to dose of exposure for a given population (e.g. alcohol intake and risk of all-cause mortality). This makes sense for decision makers as it is expected to contain more information and be of higher clinical value. The abstract reporting of SR of dose-response meta-analysis (SR-DRMA) is therefore expected to be more informative than traditional meta-analysis. Knowing about the abstract reporting is useful for further studies and helpful to form the standard reporting checklist specific to DRMA [10]. Nevertheless, there were currently no researches investigated the abstract reporting of SR-DRMAs.

We conducted a literature survey of the published SR-DRMAs of the abstract reporting from 2011 to 2017 to investigate the reporting quality of the abstract and to determine potential influential factors for the quality.

## Method

### Data source and search strategy

This study was reported according to the PRISMA statement [11]. We searched Medline, Embase, and Wiley online Library for SR-DRMAs published from 1st -January-2011 to 31st-December − 2015 and then updating the searching to 31st-July-2017. We limited the time range because there were little DRMAs published before 2011. A combination of keywords and index terms related to dose-response meta-analysis, meta-analysis of cohort studies, meta-analysis of prospective studies, meta-analysis of observational studies and non-linear meta-regression was used after discussed with four core investigators with expertise in literature search. We did not search the grey literature and no limitations were made on the language. The full search strategy was provided in Additional file 1.

### Eligible criteria and study selection

We included published aggregate (in contrast to individual participant data) SR-DRMAs with binary outcomes in the biomedical field. The definition of SR-DRMAs has been clarified in the background [9]. Traditional meta-regression analysis and survival analysis were not considered as DRMA here. There was no limitation on the population, exposure/intervention, health issues, as well as study design in each SR-DRMA. We focused on binary outcomes because there currently were very few SR-DRMAs with continuous outcomes available and the results reporting of them were of more flexible (for example, it allows zero-reference for relative difference and non-zero-reference for absolute difference) [12]. We did not consider unpublished article and conference abstract because such types of publications generally not peer reviewed.

Study selection was conducted independently by two investigators (XC and LY). We first excluded duplicates by reference management software (Endnote X7). We subsequently reviewed the titles and abstracts of each citation and made a decision regarding its appropriateness for inclusion. Full texts of potentially eligible articles were further assessed by the two investigators independently and any disagreement was solved by consensus. We calibrated the decisions of the two investigators by the “notes” and “find duplicate” functions in Endnote software and those with same decisions were identified as duplicates.

### Data extraction

For each SR-DRMA, information was extracted separately including year of publication, region of first author (by affiliation), number of authors, word count of the abstract, structure of abstract, journals (journal name, scope) and funding information. Two screeners (XB and HXH) extracted the data independently and cross-checked the extracted information.

### Assessment of abstract reporting

The reporting of abstracts was assessed using the PRISMA for Abstracts [7], which includes a checklist pertaining to each section of the abstract, including “title”, “objective”, “methods”, “results”, “conclusion” and “other information” with totally 12 items. In order to make it more suitable for SR-DRMA, we slightly modified the checklist by adding two additional items (in prior) that most of the Cochrane systematic reviews contained: (1) methods of combining dose-response data (Item 6); (2) description and evaluation of quality (risk of bias) of included study (Item 8). We also predefined the type of results of main outcomes as linear or dose-specific absolute risk (AR) or relative risks (RR) for item “results of main outcomes”. This modification may have some impact on the total score and data analysis.

The modified PRISMA for Abstract therefore contains 14 items (Additional file 1: Table S1), with each item corresponding to three response options: ‘Yes’, ‘No’, or ‘Unclear’. We assign 1 score for the item for “Yes” while 0 for “no” or “unclear” [13]. Then, the quality score of the abstract ranges from 0 to 14 and an abstract with higher score was regarded as better reporting.

Two researchers (XB and HXH) independently assessed the quality of the abstracts using the modified PRISMA for Abstracts checklist. Any disagreement was solved by consensus after the assessment. The inter-rater correlation was calculated using the kappa (κ) statistics as a measurement of the degree of agreement [14].

### Data analysis

Descriptive statistics were used to summarize the basic characteristics. In detail, we used the median and quartile to describe the overall score distribution and the frequency and proportion for the categorical data. Adherence rate (AR, $\mathrm{AR}=\frac{\mathrm{n}}{\mathrm{N}}\times 100\%$) and 95% confidence interval (CI) were used to reflect the degree of compliance of each item. Here n is the number of SR-DRMAs adhere the requirement of certain item while N is the total SR-DRMAs included. We divided the adherence rate of each item into three levels: well complied (met by 80% or above), moderately complied (met by 50 to 79%), and poorly complied (met by less than 50%) [15]. It should be noted that this kind of division is arbitrary.

We used the total score to reflect the quality of the reporting. This is reasonable since it was widely applied in such types of researches [16, 17]. In order to investigate potential factors related to the quality of the abstract reporting, we pre-specified the following 5 variables to regress with the total quality score: year of publication, region of first author (Asia-pacific, European, America), number of authors, word count of the abstract (> 250 vs. ≤ 250), and funding (yes vs. no). We choose these factors because we aimed to see if the quality of abstract reporting was increased by years, differed by regions, and improved by more authors, more words as well as financial supporting. Of which, previous literatures have suggested that these variables may influence the abstract reporting [18, 19].

We used weighted least square linear regression analysis to modeling the relationship between the 5 variables and the total score, by considering the potential heteroscedasticity on parameter estimation [20]. We employed the robust variance that treats each journal as a cluster to address correlations of the reporting quality of SR-DRMAs published in the same journal. The generalized linear model equation (GEE) was conducted as sensitivity analysis. Data analyses were performed by STATA statistical software (Version 12.0, College Station, TX) and *P* <  0·05 was treated as statistical significant.

## Results

**Fig. 1:**
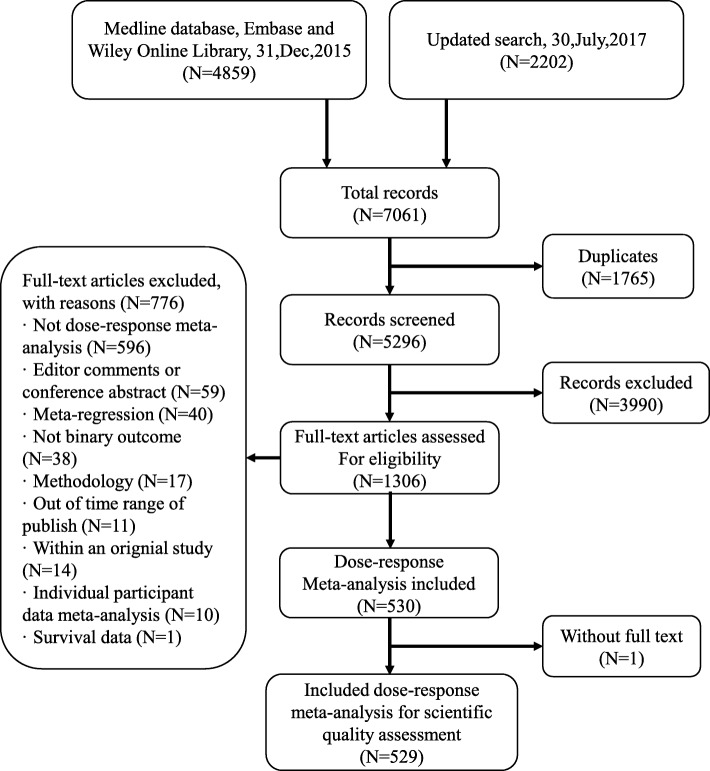
The flow chart of literature screen

The initial literature search retrieved 7061 records. After removing 1765 duplicates and 3990 clearly irrelevant records, full-text papers of the remaining 1306 records were identified for final assessment. Among the 1306 records, 776 were excluded by the following reasons: not dose-response meta-analysis (*n* = 596), not binary outcome (*n* = 38), editor comments or conference abstract (*n* = 59), meta-regression analysis (*n* = 40), methodology study (*n* = 17), out of time range of publish (*n* = 11), meta-analysis contained within an original study (*n* = 14) or, individual participant data (*n* = 10) and survival data (*n* = 1). Finally, a total of 529 SR-DRMAs were included in this cross-sectional analysis (Fig. 1).

### General characteristics

The 529 SR-DRMAs were published in 174 different academic journals. Most of which were in specialist (disease-specific) journal (*n* = 365, 69.0, 95% CI: 64.9, 72.9%), followed by general journal (*n* = 119, 22.50, 95% CI: 19.0, 26.3%) and epidemiology or public health journal (*n* = 45, 8.51, 95% CI: 6.10, 10.90%). Among the 529 abstracts, the median number of word count was 245 [first quartile, third quartile: 212, 267.5], of which 307 (58.0, 95% CI: 53.7, 62.3%) were 250 or less. Most of the SR-DRMAs used structured abstract (*n* = 338, 63.9, 95% CI: 59.6, 68.0%). Table 1 presents the details of basic characteristics of the included DRMAs.

**Table 1:** General characteristics of published DRMAs of the abstract

Category by items	All Publications (*N* = 529)
Word count	245 (212 to 267.5)
≤250	307 (58.0%)
> 250	222 (42.0%)
Structured abstract	
Yes	338 (63.9%)
No	191 (36.2%)
Number of authors [median (IQR)]	6 (4 to 8)
≤ 4	171 (32.3%)
5 ~ 6	125 (23.6%)
7 ~ 8	153 (28.9%)
> 8	80 (15.1%)
Year of publish	
2011	35 (6.6%)
2012	44 (8.3%)
2013	56 (10.6%)
2014	117 (22.1%)
2015	120 (22.7%)
2016	85 (16.0%)
2017 (up to July-31)	72 (13.6%)
Journals (*n* = 174 for journal numbers)	
Specialist journal (disease-specific)	365 (69.0%)
General journal (all diseases)	119 (22.5%)
Epidemiology or public health	45 (8.5%)
Region of first author	
Asian	350 (66.2%)
European	129 (24.4%)
America	47 (8.9%)
Australia	3 (0.6%)
Funding	
Yes	337 (63.7%)
No	54 (10.2%)
Not reported	138 (26.1%)

*IQR* interquartile range

Among these SR-DRMAs, 350 (66.2, 95% CI: 62.0, 70.2%) were conducted by authors (first author) from Asian region, 129 (24.4, 95% CI: 20.8, 28.3%) from Europe, 47 (8.9, 95% CI: 6.6, 11.6%) from North America, and 3 (0.6, 95% CI: 0.1, 1.6%) from Australia. 328 (67.7, 95% CI: 63.5, 71.6%) of the 529 SR-DRMAs have more than 4 authors and the median of the authors number was 6 [first quartile, third quartile: 4, 8]. Most of the SR-DRMAs got funding supports (*n* = 337, 63.7, 95% CI: 59.4, 67.8%). Within the SR-DRMAs being funded (*n* = 337), 336 were supported by government and one was supported by the company.

### Adherence rate of each reporting item

**Fig. 2:**
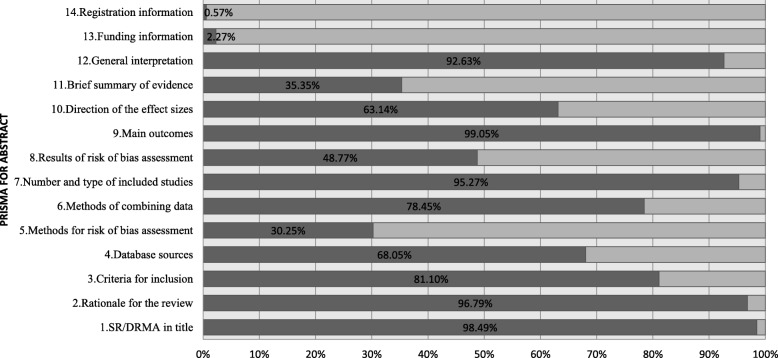
The adherence rate of single item of the abstract. Adherence rate indicates the proportion of SR-DRMAs meet the requirement of the item

The two authors achieved a reasonable consistency on assessing the abstract reporting with each item had a κ value over 0.75 and the overall item had a κ value as 0.95 (Additional file 1: Table S2). Generally, 6 out of 14 items were well complied, 3 were moderately complied, and 5 were poorly complied. The adherence rate of items reported for the PRISMA checklist was listed in Fig. 2.

The title section contains 1 item, which was presented as “identify the report as a systematic review, dose-response meta-analysis, or both”. This was compiled by most of the SR-DRMAs (AR = 98.5, 95% CI: 97.1, 99.2%). The objective section indicated the rationale for the review, and was compiled by 96.8% of the SR-DRMAs (AR = 96.8, 95% CI: 94.9, 98.1%).

For the methods section, there were 4 items appointed, including clarifying the criteria for inclusion (AR = 81.1, 95% CI: 77.5, 84.3%), database sources (AR = 68.1, 95% CI: 63.9, 72.0%), methods for risk of bias assessment (30.3, 95% CI: 26.4, 34.4%), and methods of combining data (78.5, 95% CI: 74.7, 81.9%). One item in this section was well complied, 2 were moderately complied, while 1 was poorly complied.

The results section contains 4 items, which referred to clarify number and type of included studies (AR = 95.3, 95% CI: 93.1, 96.9%), results of risk of bias assessment (AR = 48.8, 95% CI: 44.4, 53.1%), main outcomes (AR = 99.1, 95% CI: 97.8, 99.7%), and direction of the effect sizes (AR = 63.1, 95% CI: 58.9, 67.3%). Of which, 2 items were well complied, 1 was moderately complied, while 1 was poorly complied.

For discussion section of the 2 items, 1 was well complied [Brief summary of evidence (AR = 35.4, 95% CI: 31.3, 39.6%)] and the other was moderately complied [General interpretation (AR = 92.6, 95% CI: 90.1, 94.7%)].

For other information, only 2.3% of the SR-DRMAs reported the funding information (AR = 2.3, 95% CI: 1.2, 3.9%) and 0.6% reported the registration information (AR = 0.6, 95% CI: 0.1, 1.6%) in the abstract.

### Risk factors for reporting quality of abstract

**Fig. 3:**
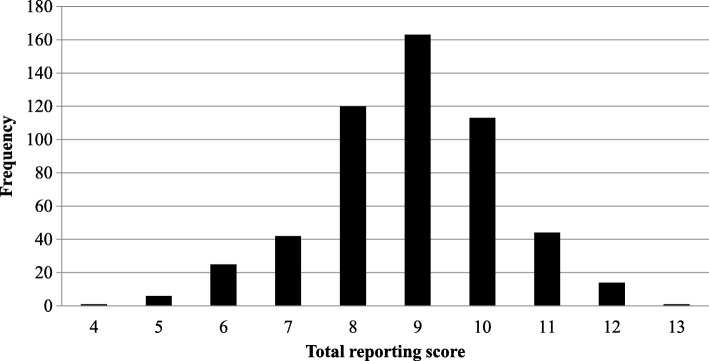
The distribution of total quality score. X-axis is the total quality score and the Y-axis is the number of SR-DRMAs under the quality score

Figure 3 presents the overall quality score of abstracts. The scores ranged from 4 to 13 with a median value of 9 (first quartile, third quartile: 8, 10). Our regression analysis showed that, after adjusted for clustering on journal, year of publication [2013 vs. 2011 (estimated ß = − 0.55; 95% CI: − 1.10, 0.00; *P* = 0.048); 2017 vs. 2011 (estimated ß = − 0.93; 95% CI: − 1.40, − 0.47; *P* <  0.001)] was adversely associated with reporting quality, while the word count of abstracts [> 250 vs. ≤ 250 (estimated ß = 0.31; 95% CI: 0.02, 0.61; *P* = 0.039)] was positively associated with the reporting quality. There were no obvious relationships between regions of first author [Asia Pacific vs. European (estimated ß = − 0.08; 95% CI: − 0.37, 0.22; *P* = 0.612); America vs. European (estimated ß = − 0.07; 95% CI: − 0.61, 0.46; *P* = 0.785)], financial support or not (estimated ß = − 0.20; 95% CI: − 0.43, 0.02; *P* = 0.077) and abstract reporting (Table 2).

**Table 2:** Multivariate regression analysis of risk factors for abstract reporting quality

Influence factors	Estimated β (95% CI)			
	WLSLR	*P*-value	GEE	*P*-value
No. of authors				
≤ 4	Reference		Reference	
5 ~ 6	−0.28 (−0.57, 0.00)	0.054	−0.36 (− 0.65, − 0.06)	0.017
7~ 8	− 0.11 (− 0.40, 0.18)	0.450	− 0.13 (− 0.40, 0.14)	0.350
> 8	− 0.03 (− 0.39, 0.33)	0.890	−0.12 (− 0.49, 0.25)	0.516
Year of publication				
2011	Reference		Reference	
2012	−0.30 (−0.76, 0.17)	0.211	−0.38 (− 0.84, 0.08)	0.105
2013	−0.55 (−1.10, 0.00)	0.048	− 0.58 (−1.10, − 0.06)	0.028
2014	−0.12 (−.061, 0.36)	0.616	− 0.14 (− 0.59, 0.31)	0.546
2015	− 0.37 (− 0.89, 0.15)	0.166	−0.39 (− 0.85, 0.07)	0.100
2016	−0.45 (− 0.97, 0.06)	0.084	−0.49 (− 0.96, − 0.02)	0.041
2017	−0.93 (− 1.40, − 0.47)	< 0.001	−0.97 (− 1.43, − 0.51)	< 0.001
Region				
European	Reference		Reference	
Asia Pacific	−0.08 (− 0.37, 0.22)	0.612	− 0.11 (− 0.38, 0.16)	0.412
America	− 0.07 (− 0.61, 0.46)	0.785	−0.03 (− 0.59, 0.53)	0.917
Funding				
No	Reference		Reference	
Yes	−0.20 (−0.43, 0.02)	0.077	−0.30 (− 0.47, − 0.05)	0.015
Word count				
≤ 250	Reference		Reference	
> 250	0.31 (0.02, 0.61)	0.039	0.28 (0.03, 0.54)	0.027

*WLSLR* weighted least square linear regression;

*GEE* generalized estimating equation

The results of sensitivity analysis showed robust results for year of publication [2013 vs. 2011 (estimated ß = − 0.58; 95% CI: − 1.10, − 0.06; *P* = 0.028); 2016 vs. 2011 (estimated ß = − 0.49; 95% CI: − 0.96, − 0.02; *P* = 0.041); 2017 vs. 2011 (estimated ß = − 0.97; 95% CI: − 1.43, − 0.51; *P* <  0.001)], word number of abstract [> 250 vs. ≤ 250 (estimated ß = 0.28; 95%CI: 0.03, 0.54; *P* = 0.027)], and region of first author [Asia Pacific vs. European (estimated ß = − 0.11; 95% CI: − 0.38, 0.16; *P* = 0.412); America vs. European (estimated ß = − 0.03; 95% CI: − 0.59, 0.53; *P* = 0.917)] with reporting quality. However, unstable results were observed. In GEE model, number of authors [5 to 6 vs. 4 or less (estimated ß = − 0.36; 95% CI: − 0.65, − 0.06; *P* = 0.017)], studies with financial support (estimated ß = − 0.30; 95% CI: − 0.47, − 0.05; *P* = 0.015) were adversely associated with the abstract reporting (Table 2).

## Discussion

In this article, we conducted a literature survey on the reporting of the abstract of published SR-DRMAs and we found that the abstract reporting was suboptimal for these SR-DRMAs. The limitations of the abstract reporting mainly embodied in the section of methods, results, and the other information (e.g. registration). In particular, we observed that most of the SR-DRMAs failed to describe the methods for risk of bias assessment and the results of bias assessment, and few SR-DRMAs reported the funding and registration information in the abstract.

Our regression analysis revealed that year of publication was adversely associated with the quality of abstract reporting while the word number of abstract was positively associated with the reporting. We also observed unstable, adverse relationship between number of authors, financial support and reporting quality of abstract. Consistent results suggested no obvious relationship between region of first author and quality of abstract reporting. A potential reason for the relationship between word count and the reporting quality was that authors have more space to describe the results. In contrast, when there was word count limits, review authors may remove those contexts that they think less important and as a result make the abstract less informative. Academic journals may consider to improve the word count limits of the abstract, especially those limited word count as less than 250.

The methods and results reporting are particularly important for a well-organized abstract, which summarizes the design, conduction, analysis and findings of the researches. In our survey, the reporting of methods and results were worrisome. There may be some connections between the abstract reporting and the full-text reporting because abstract depends on the work of what it is summarizing from the full-text. In our previous survey, we observed that the reporting on the methods and results for the full-text of SR-DRMAs were uninformative [21] (Additional file 1: Figure S1). These findings highlighted the importance of the reporting of abstract that it may partly reflect the quality of full-text reporting, and thus review authors should also focus on the completeness of SR-DRMA itself. For systematic review, risk of bias assessment is the essential part and further review authors should report such information, regardless in the abstract and the full-text. There were small amount (less than 1/3) of SR-DRMAs described the methods to access risk of bias of included studies in the abstract while a higher proportion (about 1/2) described the results of risk of bias. It was interesting that more SR-DRMAs reported the combined effect sizes (99.05%) than the combining methods (78.45%). We hypothesized that review authors tend to focus on the results rather than the methods.

In our survey, very few SR-DRMAs reported the financial and registration information in the abstract. Indeed, most of the SR-DRMAs reported these information in the full-text (Additional file 1: Figure S1). Such information was important for decision makers and systematic review producers. Previous literature has demonstrated that substantial financial bias may exaggerate the efficiency while cover up the harms of clinical trial [22]. In many academic journals, funding and registration information were required at the full text while no mandatory for abstract. We recommended SR-DRMA authors and academic journals diligent such in formation in the abstract.

In this survey, we did not observe obvious improvement of the reporting quality of SR-DRMAs over the years from 2011 to 2017, though the PRISMA for abstract was released in 2013 [7]. This finding is similar to a previous research that investigated the abstract reporting of randomized controlled trials [23]. In that review, Chhapola et al. used the Consolidated Standards of Reporting Trials (CONSORT) for Abstract extension to assess the abstract reporting and their research showed insignificant change before and after the publication of the CONSORT abstract guideline [23].

There were several strengthens of current research. This is the first literature survey on the abstract reporting of published SR-DRMAs. We included almost all of the SR-DRMAs published during the past 7 years for the analysis. Our findings have directive significance for systematic reviewers of SR-DRMA and guideline developers for abstract reporting. Moreover, in an attempt to ensure the validity of quality assessment, we estimated the inter-rater correlation of each item of the judgment. The results suggested a good consistency of the assessment between the two assessors. We also employed the weighted least square regression and the robust variance to achieve credible parameter estimation. Sensitivity analyses for most of the results were stable.

Several limitations should be highlighted. The major limitation in this survey was that we used the modified PRISMA for abstract checklist to access the reporting quality by adding two additional items. Such arbitrary modification may have some influence on the total quality score and the regression analysis. The credibility and validity of the modified checklist needs to be verified. Second, we only assessed the SR-DRMAs of aggregate data and binary outcomes. The findings of our research may be not suitable for SR-DRMAs based on individual participant data and those with continuous outcomes. Third, some of the results in our regression analysis were instable. For example, the relationship between number of authors, financial support and reporting quality of abstract are inconsistent in sensitivity analysis. These two results should be treated with caution.

## Conclusions

The abstract reporting of SR-DRMAs is suboptimal. Substantial effort is needed to improve the reporting, especially for the reporting of the methods and results. More words number may benefit for the abstract reporting and at least 250 words were recommended for SR-DRMAs. Given that the reporting of abstract largely depends on the reporting and conduct of the SR-DRMA, review authors should also focus on the completeness of SR-DRMA itself.

## Additional files

Additional file 1: Search Strategy. **Table S1.** Modified PRISMA for Abstract. **Table S2.** Kappa (κ) statistics for the inter-rater correlations. **Figure S1.** Comparison of abstract reporting and full-text reporting. (DOCX 73 kb)

Additional file 2: The raw data for quality of abstract reporting. (XLSX 39 kb)

## Data Availability

The dataset supporting the conclusions of this article is included within the article and its Additional files 1 and 2.

## Abbreviations

AR: Absolute risk
AR: Adherence rate
CONSORT: Consolidated Standards of Reporting Trials
DRMA: Dose-response meta-analysis
GEE: Generalized linear model equation
PRISMA: Preferred Reporting Items for Systematic Reviews and Meta-Analyses
RR: Relative risks
SR-DRMA: Systematic reviews of dose-response meta-analysis
SRs: Systematic reviews
