# Supplementary material for: Assessment of the abstract reporting of systematic reviews of dose-response meta-analysis: a literature survey
Source: BMC Med Res Methodol. 2019 Jul 15;19:148. doi: 10.1186/s12874-019-0798-5 (PMC6631883; doi:10.1186/s12874-019-0798-5)

**Search Strategy**

**Ovid MEDLINE(R) In-Process & Other Non-Indexed Citations and Ovid MEDLINE(R) 1946 to Present (2011 to** **2015-12-31)**

| **Items** | **Searches** | **Results** | **Search Type** |
| --- | --- | --- | --- |
| 1 | Dose Response Relationship*/exp {No Related Terms} | 473 | Basic |
| 2 | Dose-response association*/exp {No Related Terms} | 9478 | Basic |
| 3 | Dose-effect*/exp {No Related Terms} | 6287 | Basic |
| 4 | or/1-3 | 16162 | Advanced |
| 5 | Meta-analysis/exp {No Related Terms} | 19789 | Basic |
| 6 | ''Pool? Analysis'' {No Related Terms} | 8322 | Basic |
| 7 | Systematic review*/exp {No Related Terms} | 15451 | Basic |
| 8 | or/5-7 | 39299 | Advanced |
| 9 | 4 and 8 | 120 | Advanced |
| 10 | (''dose-response meta-analysis'' or ''non-linear meta-regression'' or ''meta-analysis of prospective studies'' or ''meta-analysis of cohort studies'' or ''meta-analysis of observational studies'').tw,ot. | 1613 | Advanced |
| 11 | 9 or 10 | 1730 | Advanced |
| 12 | animals/ not (Humans/ and Animals/) {No Related Terms} | 10028 | Basic |
| 13 | 11 not 12 | 1730 | Advanced |
| **14** | **13 and 2011:2015.(sa_year).** | **1381** | **Advanced** |

**Embase database**

| **No.** | **Query** | **Results** |
| --- | --- | --- |
| #19 | **#18** AND [embase]/lim | **2,474** |
| #18 | **#17** AND (**'evidence based medicine'**/de OR **'human'**/de OR **'meta analysis'**/de OR **'meta analysis (topic)'**/de OR **'systematic review'**/de) AND (**2011**:py OR **2012**:py OR **2013**:py OR **2014**:py OR **2015**:py) | **2,598** |
| #17 | **#10** OR **#16** | **6,450** |
| #16 | **#11** OR **#12** OR **#13** OR **#14** OR **#15** | **1,832** |
| #15 | **'meta-analysis of observational studies'** | **1,008** |
| #14 | **'meta-analysis of cohort studies'** | **290** |
| #13 | **'meta-analysis of prospective studies'** | **375** |
| #12 | **'non-linear meta-regression'** | **1** |
| #11 | **'dose-response meta-analysis'** | **262** |
| #10 | **#5 AND #9** | **4,788** |
| #9 | **#6 OR #7 OR #8** | **164,355** |
| #8 | **'systematic review'/exp** | **101,893** |
| #7 | **'pool? analysis'** | **17** |
| #6 | **'meta analysis'**/exp | **102,438** |
| #5 | **#1** OR **#2** OR **#3** OR **#4** | **372,838** |
| #4 | **'dose-effects'** | **1,534** |
| #3 | **'dose-effect'**/exp | **371,631** |
| #2 | **'dose-response association'** | **485** |
| #1 | **'dose response relationship'**/exp | **371,631** |

**Wiley Online Library (2011~2015)** *1004 results were presented*

(''dose-response meta-analysis'' or ''non-linear meta-regression'' or ''meta-analysis of prospective studies'' or ''meta-analysis of cohort studies'' or ''meta-analysis of observational studies'') in Abstract NOT animal* in All Fields between years 2011 and 2015

**Table S1.** Modified PRISMA for Abstract

| **Title** | 1. Identify the report as a systematic review, meta-analysis, or both. |
| --- | --- |
| **Objectives** | 2. The research question including components such as participants, interventions, comparators, and outcomes |
| **Methods** | 3. Study and report characteristics used as eligibility criteria for inclusion |
|  | 4. Key databases searched and search dates |
|  | 5. Methods of assessing risk of bias |
|  | 6. Methods of combining dose-response data |
| **Results** | 7. Number and type of included studies and participants |
|  | 8. Description and evaluation of quality (risk of bias) of included study |
|  | 9. Synthesis results for main outcomes (e.g. linear results, non-linear results), preferably indicating the number of studies and participants for each. If meta-analysis was done, include summary measures and confidence intervals. |
|  | 10. Direction and size of the effect |
| **Discussion** | 11. Brief summary of strengths and limitations of evidence (e.g. inconsistency, imprecision, indirectness, or risk of bias, other supporting or conflicting evidence) |
|  | 12. General interpretation of the results and important implications |
| **Other** | 13. Primary source of funding for the review |
|  | 14. Registration number and registry name |

**Table S2.** Kappa (κ) statistics for the inter-rater correlations

| **Items** | **Rater 1** | **Rater 2** | | **Kappa** |
| --- | --- | --- | --- | --- |
|  |  | No | Yes |  |
| ***Item 1*** | No | 8 | 0 | 1.000 |
|  | Yes | 0 | 521 |  |
| ***Item 2*** | No | 17 | 4 | 0.802 |
|  | Yes | 4 | 504 |  |
| ***Item 3*** | No | 103 | 1 | 0.947 |
|  | Yes | 8 | 417 |  |
| ***Item 4*** | No | 169 | 0 | 1.000 |
|  | Yes | 0 | 360 |  |
| ***Item 5*** | No | 349 | 13 | 0.792 |
|  | Yes | 33 | 134 |  |
| ***Item 6*** | No | 123 | 6 | 0.938 |
|  | Yes | 6 | 394 |  |
| ***Item 7*** | No | 25 | 0 | 0.960 |
|  | Yes | 2 | 502 |  |
| ***Item 8*** | No | 261 | 10 | 0.864 |
|  | Yes | 26 | 232 |  |
| ***Item 9*** | No | 5 | 0 | 1.000 |
|  | Yes | 0 | 524 |  |
| ***Item 10*** | No | 207 | 9 | 0.891 |
|  | Yes | 19 | 294 |  |
| ***Item 11*** | No | 334 | 8 | 0.908 |
|  | Yes | 14 | 173 |  |
| ***Item 12*** | No | 48 | 5 | 0.78 |
|  | Yes | 16 | 460 |  |
| ***Item 13*** | No | 517 | 0 | 1.000 |
|  | Yes | 0 | 12 |  |
| ***Item 14*** | No | 526 | 0 | 1.000 |
|  | Yes | 0 | 3 |  |
| ***Overall*** | No | 2692 | 56 | 0.947 |
|  | Yes | 128 | 4530 |  |

**Figure S1.** Comparison of abstract reporting and full-text reporting


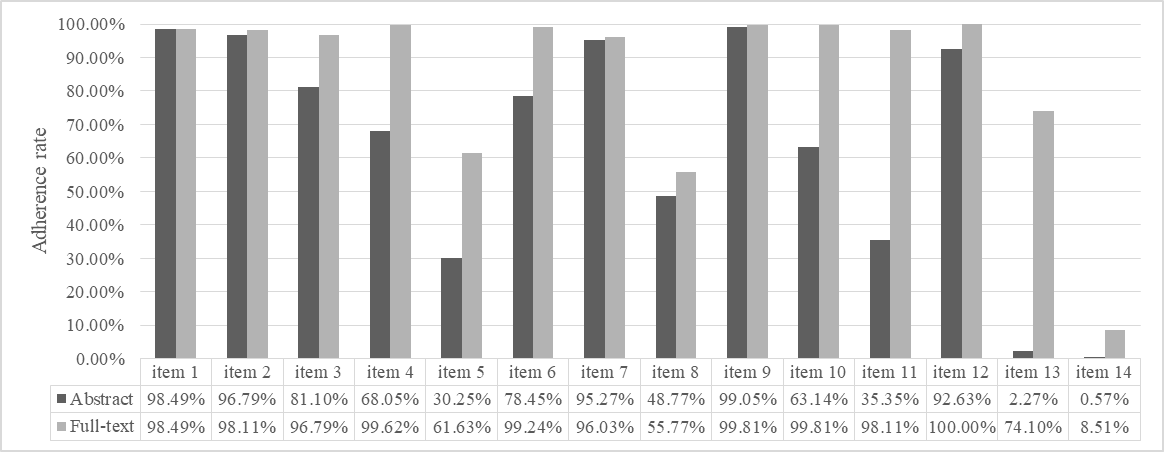

Supplement: Supplementary file 1 — Search Strategy. Table S1. Modified PRISMA for Abstract. Table S2. Kappa (κ) statistics for the inter-rater correlations. Figure S1. Comparison of abstract reporting and full-text reporting. (DOCX 73 kb) [file 12874_2019_798_MOESM1_ESM.docx]
